# Biochemical Characterization and Cellular Effects of CADASIL Mutants of NOTCH3

**DOI:** 10.1371/journal.pone.0044964

**Published:** 2012-09-18

**Authors:** He Meng, Xiaojie Zhang, Genggeng Yu, Soo Jung Lee, Y. Eugene Chen, Igor Prudovsky, Michael M. Wang

**Affiliations:** 1 Department of Neurology, University of Michigan, Ann Arbor, Michigan, United States of America; 2 Department of Internal Medicine, University of Michigan, Ann Arbor, Michigan, United States of America; 3 Department of Molecular & Integrative Physiology, University of Michigan, Ann Arbor, Michigan, United States of America; 4 Center for Molecular Medicine, Maine Medical Center Research Institute, Scarborough, Maine, United States of America; 5 Neurology Service, VA Ann Arbor Healthcare System, Ann Arbor, Michigan, United States of America; National Institute of Health, United States of America

## Abstract

Cerebral Autosomal Dominant Arteriopathy with Subcortical Infarcts and Leukoencephalopathy (CADASIL) is the best understood cause of dominantly inherited stroke and results from NOTCH3 mutations that lead to NOTCH3 protein accumulation and selective arterial smooth muscle degeneration. Previous studies show that NOTCH3 protein forms multimers. Here, we investigate protein interactions between NOTCH3 and other vascular Notch isoforms and characterize the effects of elevated NOTCH3 on smooth muscle gene regulation. We demonstrate that NOTCH3 forms heterodimers with NOTCH1, NOTCH3, and NOTCH4. R90C and C49Y mutant NOTCH3 form complexes which are more resistant to detergents than wild type NOTCH3 complexes. Using quantitative NOTCH3-luciferase clearance assays, we found significant inhibition of mutant NOTCH3 clearance. In coculture assays of NOTCH function, overexpressed wild type and mutant NOTCH3 significantly repressed NOTCH-regulated smooth muscle transcripts and potently impaired the activity of three independent smooth muscle promoters. Wildtype and R90C recombinant NOTCH3 proteins applied to cell cultures also blocked canonical Notch fuction. We conclude that CADASIL mutants of NOTCH3 complex with NOTCH1, 3, and 4, slow NOTCH3 clearance, and that overexpressed wild type and mutant NOTCH3 protein interfere with key NOTCH-mediated functions in smooth muscle cells.

## Introduction

Cerebral Autosomal Dominant Arteriopathy with Subcortical Infarcts and Leukoencephalopathy (CADASIL) is an inherited arteriopathy marked by migraine headaches, premature stroke, and vascular dementia [1], [2], [3]. CADASIL is caused by mutations in *NOTCH3* [4], which encodes an evolutionarily conserved transmembrane receptor [5], [6], [7], [8], [9], [10], [11]. Almost all mutations described to date result in a change in the number of cysteines in the extracellular domain of the protein, which has led to the hypothesis that abnormalities in NOTCH3 secondary structure cause the arterial pathology seen in CADASIL [12], [13], [14], [15], [16]. Tissues from patients with CADASIL display damage to smooth muscle cells of small arteries and arterioles, characterized by cell-cell separation and cell loss, accumulation of granular osmiophilic material (GOM) around cells, fibrosis, and marked accumulation of NOTCH3 protein in the media [17], [18], [19], [20]. In spite of this detailed description of pathology, a comprehensive view of the molecular and cellular changes induced by NOTCH3 excess has yet to emerge.

Notch signal transduction plays an important role in the development and homeostasis of blood vessels. Notch or Notch ligand knockout and activated Notch transgenic mice display marked vascular phenotypes, ranging from subtle arterio-venous specification disorders to severe angiogenic failure [21], [22], [23], [24], [25], [26], [27], [28]. Notch also plays a role in homeostatic control and responses of the mature vasculature to disease [29], [30], [31], [32]. Although a majority of work has focused on Notch in endothelial cells, smooth muscle Notch has also been shown to regulate cell growth, death, and differentiation [33], [34], [35]. The profound effect of the Notch system on blood vessels suggests that in CADASIL, interference with normal NOTCH signaling by mutant NOTCH3 could play a major mechanistic role. Recently, NOTCH3 protein has been shown to form multiprotein complexes [36], [37]; CADASIL mutations in NOTCH3, moreover, have been shown to slow the degradation of NOTCH3 [38]. These studies suggest that smooth muscle cells in CADASIL accumulate NOTCH3 due to mutations in the protein and implicate protein accumulation as a component of the pathological process. However, to date, the cellular effects of NOTCH3 overexpression have not been addressed. The purpose of this study was to investigate whether excessive NOTCH3 is capable of forming complexes with other NOTCH proteins. We discovered that NOTCH3 could bind tightly to multiple vascular NOTCH ectodomains and strongly inhibited Notch function. Since NOTCH regulates crucial smooth muscle genes, we tested whether the overexpression of NOTCH3 affects the regulation of genes known to regulate smooth muscle homeostasis.

## Materials and Methods

### Materials

Cell culture products were obtained from Invitrogen (Carlsbad, CA) unless noted; puromycin was purchased from Alexis (San Diego, CA). All other chemicals were obtained from Sigma (St. Louis, MO) unless indicated otherwise.

### Construction of cDNA Clones Encoding Human NOTCH3

**Figure 1:**
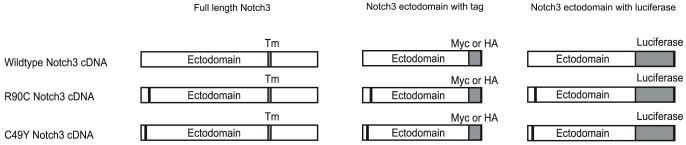
Constructs used in this study. Schematic representation of constructs used in this study, as described in the methods. The positions of the mutations examined in the present study are indicated by small dark rectangles. Wildtype and mutant constructs were all cloned into pCMV-Sport6 (Invitrogen). Tm  =  transmembrane domain of NOTCH3, which is deleted in tagged constructs. The constructs were prepared in pCMV-Sport6 (Invitrogen). Tagged NOTCH3 ectodomain constructs were prepared by replacing the C-terminal 3 kb of NOTCH3 with either an HA or myc tag or with the full coding sequence of firefly luciferase. The HA and myc tagged proteins run on SDS gels at an apparent molecular weight of 175 kDa.

Please see Figure 1 for representation of constructs. We constructed a clone for NOTCH3 in the expression vector pCMV-Sport6 (Invitrogen, Carlsbad, CA) by using publicly available cDNA clones and RT-PCR from human brain RNA [39]. We created two mutants of NOTCH3 (R90C and C49Y; mutation sites represented by black bars) by PCR based mutagenesis [40]. Epitope tagged ectodomain fragments were created by insertion of oligonucleotides encoding the appropriate tags into the NotI site of the human *NOTCH3* cDNA, resulting in an open reading frame that corresponds to the first 33 EGF-like repeats of the molecule. *NOTCH3*-luciferase fusions were created by splicing luciferase from pGL3 (Promega) into the same internal Not1 site and a 3′ XbaI site just beyond the end of the coding sequence. The 5′ fragment of full length mouse *NOTCH4* cDNA (a gift of Jan Kitajewski) spanning base pairs 1–4309 was cloned in frame between the EcoRI and NotI restriction sites of the pcDNA4V5zeo vector (Invitrogen) to obtain a C-terminal V5-tagged *NOTCH4* extracellular domain.

### Cell Culture

293A (Qbiogene, Irvine, CA), A7R5 rat aortic smooth muscle, and H460 human lung cancer cells were grown in DMEM supplemented with 10% FBS. 293A cell lines were generated by cotransfection using Lipofectamine 2000 (Invitrogen, Carlsbad, CA) according to the manufacturer’s protocol. DNA for transfections included a 1∶20 mixture of a puromycin resistance plasmid (Ambion, Austin, TX) and full length human NOTCH3 clones (wildtype and R90C or C49Y mutants). Lines containing empty vector were prepared as controls. Clones were isolated after two weeks of selection in puromycin (2 ug/ml). Experiments using cell lines were verified using at least one additional independent clone, and all NOTCH3 clones selected expressed equivalent amounts of protein. A7R5 cells were transfected in 48 well plates with 200 ng of total DNA and 1 ul of Lipofectamine 2000 per well. Transfection efficiencies typically ranged from 25–50% when assessed using GFP expression plasmids.

### Immunoblotting

Western blots were performed using standard methods onto PVDF membranes using commercial antibodies for NOTCH3, HA (F7), and myc (9E10) (R&D and Santa Cruz Biotechnology; 1∶2000 dilution). Detection was performed using enhanced chemiluminescence (Amersham, Pisctaway, NJ) or by using infrared conjugates of secondary antibodies (Rockland) detected by a LiCor flatbed Odyssey imager.

### Immunoprecipitation

Plasmids encoding the extracellular domain of human *NOTCH1* or *NOTCH4* (tagged with V5 [41]) and/or *NOTCH3* tagged with HA or myc epitopes were transfected into 293A cells. Cell lysates were prepared in modified RIPA buffer, sonicated, and cleared by centrifugation [42]. Supernatants were incubated with antibodies (1 ug) and then mixed with protein G agarose beads overnight. Beads were pelleted and washed with modified RIPA buffer three times.

### Detergent Extraction of Cell Lysates

Experiments were performed based on a protocol developed by Wang et al. [43]. Cell lines were plated at desired density and approximately 250 ug of protein was analyzed as follows: cells were rinsed with PBS, scraped, lysed by sonication in AO buffer (Tris 10 mM, pH 7.4, 100 mM NaCl, protease cocktail (Pierce, Rockford, IL) and sedimented at 15,000×G in an Eppendorf microfuge for 30 minutes at 4C. The supernatant was designated S1. The pellet was sonicated in A1 buffer (AO with 0.5% Triton) and pelleted as before. The supernatant was designated S2. The pellet was sonicated in A2 buffer (AO with 1% Triton and 0.5% deoxycholic acid) and recentrifuged; the supernatant was designated S3, and the pellet (S4) was resuspended in sample buffer. Fractions S1 through S4 were all resuspended in sample buffer and analyzed by Western blotting for NOTCH3 content.

### Degradation Assays

We determined the kinetics of NOTCH3 ectodomain clearance from cells by genetically fusing the NOTCH3 ectodomain to firefly luciferase. 293A cells were transfected with *NOTCH3*-luciferase fusions (see Figure 1) for 24 hours in 24 well plates. Cells were treated with 100 ug/ml cycloheximide and serum free media to arrest protein synthesis. At defined time points beginning 24 hours after cycloheximide treatment, cells were analyzed for luciferase expression. Total firefly luciferase content was normalized to initial luciferase content (time 0). The decrease in luciferase activity over time was used to determine the effects of NOTCH3 mutations on clearance of NOTCH3 ectodomain.

### Notch Signaling Assays

We used a cell co-culture system that was similar to previous studies [42], [44]. For mRNA expression assays, we transfected A7R5 cells transfected (three wells per group) with *NOTCH3* cDNA constructs with ligand expressing L cells expressing Jagged or Delta (or L cells without ligand as a control) [45]. After 24 hours, RNA from each well was prepared from cocultures, reverse transcribed, and quantified by real time PCR using beta-actin as a control. The average expression level for each group (n = 3 wells per co-culture; normalized to L cell controls) reflects target gene regulation by the Notch pathway. Finally, we determined the average levels of gene induction for three independent experiments. Primer sequences were designed to specifically measure A7R5 (rat) smooth muscle cDNA (and not mouse cDNA from ligand producing L cells). Sequences of these primers were: SM22∶5′-AAGAATGGCGTGATTCTGAGC-3′ 5′-CTGCCTTCAAGAATTGAGCC-3′. SMA: 5′- CAGACACCAGGGAGTGATGGT -3′ 5′CTTTTCCATGTCGTCCCAGT -3′. Calponin: 5′-CAGTCAGCAGGGCATGACAG-3′ 5′-AGTCATGCCAGCCTGGCTG-3′. SM-MHC: 5′- CCGGCAACGCTACGAGAT -3′ 5′- AGAAGATTTTGCTCTGCCCA -3′.

For luciferase-based assays, H460 (that express only NOTCH3) or A7R5 cells (cells which express multiple Notch receptors) were transiently transfected with an expression clone (vector or *NOTCH3* construct) in combination with the HES-luciferase reporter that reflects canonical NOTCH pathway activation [39], [46], smooth muscle actin-luciferase (a 2.5 kb promoter construct), SM22-luciferase [47], or smooth muscle myosin heavy chain-luciferase mixed with a renilla luciferase standard. In some experiments, we applied recombinant, purified NOTCH3-Fc (1 ug/ml; 3 nM) or human IgG (control) protein to cocultures of reporter-transfected A7R5 cells (without co-transfection of NOTCH3 cDNA) and ligand expressing cells. To generate a Notch-insensitive promoter control, we mutated the RBP site at −369 (from TTCCCAC to GATATCC; [48]) of the mouse SM22-luciferase reporter using PCR. The cells were allowed to recover overnight to enhance expression of the constructs. The following day, Notch was stimulated by overlaying Jagged1 or Delta1 expressing L cells (or parental L cells as a control) onto the Notch expressing monolayer [44], [45]. After 18–24 hours, luciferase activity was determined from cell lysates; the ratio of firefly to renilla luciferase represented the activation of the Notch pathway in the transfected cell pool. Reporter studies were done in 48 well plates, and cells were transfected with 100 ng of reporter luciferase constructs, 1 ng of renilla luciferase control plasmid, and 100 ng of expression constructs (or vector control).

### Recombinant NOTCH3 Protein Purification

Cell lines were prepared that stably expressed human NOTCH3 cDNA (corresponding 1,040 amino acids encoding the first 27 EGF-like repeats of NOTCH3; wild type or R90C mutant) fused to a myc tag and the Fc domain of murine IgG2 in pSec-Tag (Invitrogen). High expressing clones were selected by puromycin selection and expanded. After expansion, media was changed to OptiMEM (Invitrogen) overnight and this serum-free media was collected, frozen, pooled and passed over a protein A agarose column to affinity purify the recombinant NOTCH3 fusion. Elution of the protein was performed at pH5.0, and the eluate was immediately neutralized with Tris pH 7.4 and dialyzed against PBS. The final protein solution was composed of only two bands, a major band (NOTCH3-Fc at approximately 150 kDa) and a minor band at 25 kDa (about 20% of the total mass by silver staining). Gel purification and mass spectroscopy of the minor band revealed it to be human IgG heavy chain made by 293 cell lines and which copurified with NOTCH3 proteins. To control for the IgG component of the protein preparation, all experiments compared the NOTCH3-Fc protein with purified human IgG as a control.

### Statistical Analysis

All figures display means with standard deviations. Statistical analysis was performed using ANOVA with appropriate post hoc analysis and significance was assigned at p<0.05.

## Results

We sought to answer four questions: 1) do wildtype or mutant NOTCH3 ectodomains physically interact with NOTCH1, 3, and 4 ectodomains; 2) do mutations in NOTCH3 affect detergent solubility; 3) do mutations in NOTCH3 impair protein clearance; and 4) does NOTCH3 overexpression affect Notch regulation of smooth muscle genes?

### NOTCH3 Homo- and Heteromeric Associations are Mediated by the Extracellular Domain

**Figure 2:**
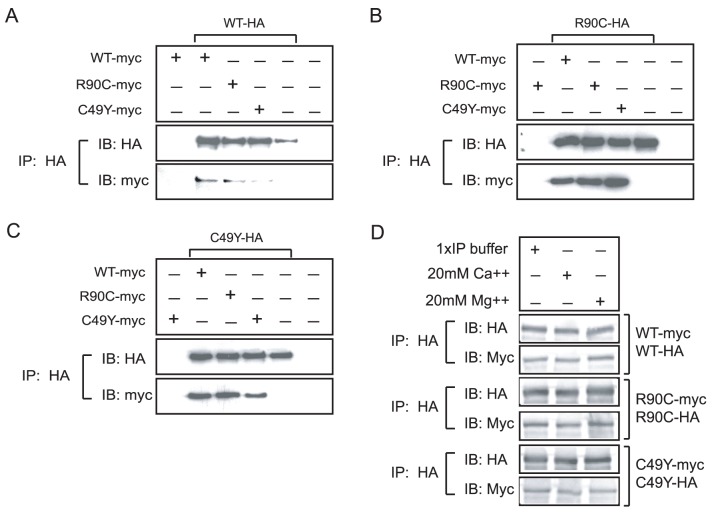
Interactions between wild-type and R90C and C49Y mutants of NOTCH3. (A–C) NOTCH3-NOTCH3 self-association is mediated by the extracellular domain. 293 cells were transiently co-transfected with cDNAs expressing the NOTCH3 extracellular domain tagged with HA (A, B, and C were transfected with WT, R90C, and C49Y, respectively) and cotransfected with myc tagged NOTCH3 ectodomain constructs as shown. The proteins were extracted after 40∼48 hours and immunoprecipitated with HA monoclonal antibody. Co-immunoprecipitates of cells cotransfected with plasmid combinations shown were analyzed by immunoblotting (IB). Quantification of protein bands from four independent experiments failed to show a significant difference in IP complexes with any of the NOTCH3 combinations. (D) Effect of divalent cations on stability of NOTCH3 homophilic interactions and the stability of NOTCH3 homophilic complexes. Coimmunoprecipitation was performed using the HA antibody on lysates of cells transfected with plasmid combinations shown on the right column. Immunoprecipitates were washed extensively with buffers containing calcium or magnesium, and then analyzed by western blotting. All of the HA and myc tagged proteins run on SDS gels at an apparent molecular weight of 175 kDa. We verified the finding of numerous groups that common epitope tags do not mediate protein interaction [64], [65], [66] (not shown). All experiments were performed more than 6 times.

Many NOTCH protein partners contain EGF-like repeats in their extracellular domains, including ligands Jagged, Delta, the Notch signal modifier thrombospondin-2 (THBS2; TSP2) [42], and LRP1 [49]; NOTCH1 has been shown to interact with itself [50], [51] and several mutants of NOTCH3 homodimerize [36], [38]. We therefore used immunoprecipitation assays to test whether NOTCH3 and additional mutants (the prototype R90C and C49Y) are capable of self-association through the EGF-repeat containing extracellular domain. 293A cells were cotransfected with two epitope tagged NOTCH3-extracellular domain constructs (depicted in Figure 1). In Figure 2A, we demonstrate that wildtype NOTCH3 ectodomain tagged with HA is capable of interacting with WT and mutant NOTCH3 ectodomains, as assessed by coprecipitation of WT-HA protein with all three myc-tagged NOTCH3 ectodomains. Negative control transfections (e.g. WT-myc protein alone or WT-HA protein alone) did not yield coprecipitated proteins with the converse tag. Mixing of NOTCH3 proteins expressed separately did not result in coprecipitated protein, indicating that interactions are favored by co-expression of proteins within the same cell (not shown). Similarly, in Figures 2B and 2C, we show that precipitation of mutant NOTCH3 proteins R90C and C49Y pulls down both wildtype and mutant proteins. Quantification of band intensities of all immunoprecipitation experiments (repeated over 8 times) demonstrated that there was no significant difference between the coimmunoprecipitations of any NOTCH3 combination. Under identical assay conditions, several additional proteins do not coprecipitate with the NOTCH3 ectodomain (see Figure S1 for an example).

Multiple EGF-like repeats in NOTCH are predicted to bind calcium [52]. Moreover, the addition of EDTA to cells expressing NOTCH proteins has been reported to stimulate proteolysis and release of the NOTCH3 extracellular domain, suggesting a conformational change resulting from calcium chelation [52]. However, immunoprecipitations prepared and washed in buffers containing either calcium or magnesium showed no difference in the stability of NOTCH3 protein complexes (Figure 2D), regardless of the presence of mutant NOTCH3 in complexes.

**Figure 3:**
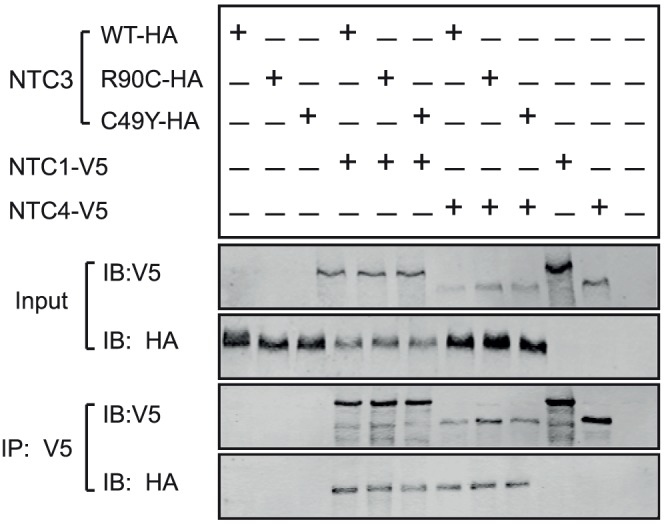
NOTCH3 ectodomain interacts with both NOTCH1 and NOTCH4 ectodomains. Interactions between NOTCH3 (WT and mutants) with NOTCH1 or NOTCH4 [41] were tested after cotransfection of plasmid combinations shown. Protein from transfected cells were evaluated by immunoblotting (input) and by immunoprecipitation with V5 monoclonal antibody followed by immunoblotting (IP:V5). Each of the NOTCH3 ectodomain constructs coprecipitated with both V5-tagged NOTCH1 or NOTCH4 proteins, which migrate with apparent molecular weights of >280 kDa and 180 kDa, respectively. All experiments were performed three times.

NOTCH1 and NOTCH4 are also expressed in blood vessels and A7R5 smooth muscle cells [53], [54], [55], [56] (data not shown). The ectodomains of these isoforms of NOTCH, like NOTCH3, are composed of a large number of EGF-like domain repeats. In cotransfection assays, we also found that wildtype and mutant NOTCH3 formed stable complexes with NOTCH1 and NOTCH4 ectodomains (Figure 3).

### CADASIL Mutations Decrease Solubility of NOTCH3 in Cell Lines

**Figure 4:**
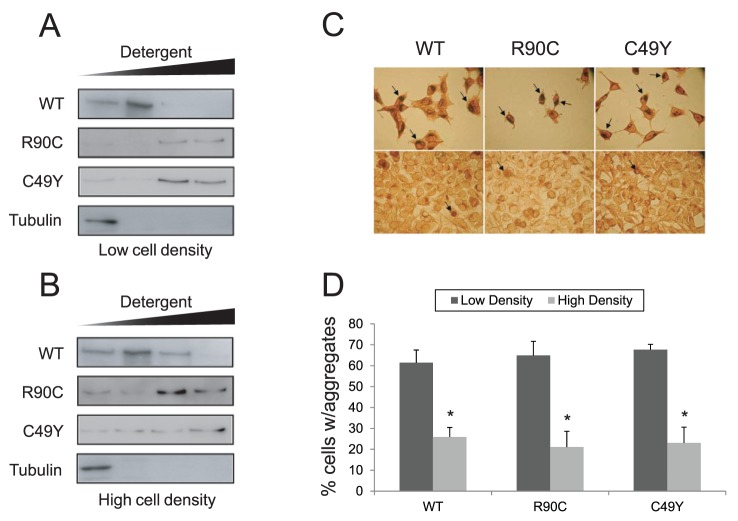
Detergent solubility and formation of inclusions of NOTCH3 expressed in cell culture. NOTCH3 complexes containing wildtype protein or mutant proteins were tested for detergent solubility. Total protein from cell lines expressing WT or mutant NOTCH3 were sequentially extracted as detailed in methods, using progressively higher strengths of detergents. Cells were grown at low density (A) or in confluent monolayers (B). While WT protein was modestly soluble, the mutant proteins were extractable only in higher detergent concentrations. NOTCH3 protein was detected using antisera from Santa Cruz. The control protein tubulin was readily extractable without detergents. Experiments were performed three times with consistent results. Cell lines were immunostained with the same antisera or with antibodies from R&D, revealing diffuse cell staining with focal accumulations in the perinuclear areas of cells expressing both wildtype and mutant proteins (C). A significant difference in the percentage of cells with perinuclear expression of the protein was notable at low cell density (D) (over 250 cells were counted for each cell line and condition. Results were reproduced three times, and significance was considered p<0.05.

We next assessed the solubility of full-length NOTCH3 wild type and mutant protein complexes from stably transfected cell lines. Full-length wildtype NOTCH3 complexes were found primarily in low concentration detergent fractions when expressed in cells plated at low density (Figure 4A). In contrast, full-length mutant NOTCH3 complexes were completely insoluble in detergent or required high concentrations of detergent for solubilization. At higher density of plating, there was a modest increase in NOTCH3 solubility (Figure 4B).

Cell lines stained for NOTCH3 demonstrated expression in the cell body and perinuclear regions (likely in the Golgi complex), reminiscent of that seen by other investigators [57], [58], [59]. Perinuclear foci were similar in wildtype and mutant full-length NOTCH3 expressing lines (Figures 4C and 4D). Plating at high cell density strongly reduced perinuclear accumulations of NOTCH3.

### NOTCH3 Mutations Delay Protein Clearance from Cells

We next tested the stability of NOTCH3 ectodomain in live cells. We fused wildtype and mutant NOTCH3 ectodomains to firefly luciferase (Figure 1), which allowed consistent and sensitive measurement of protein levels. Cells expressing these fusion proteins were treated with cycloheximide to inhibit further protein synthesis. At the end of specified periods after halting protein synthesis, cell lysates were analyzed for levels of cell-associated luciferase expression.

**Figure 5:**
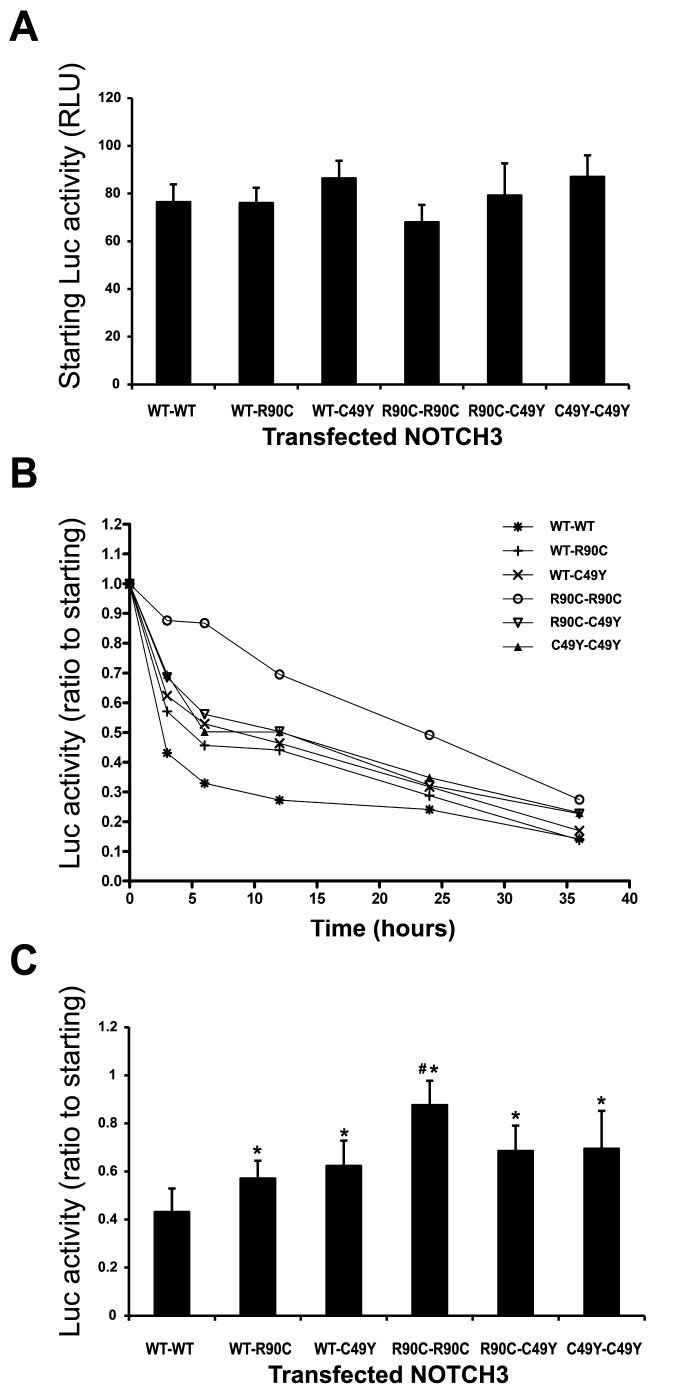
Clearance of mutant NOTCH3 proteins. 293 cells (n = 3 replicate wells per group) were transiently transfected with NOTCH3-luciferase constructs and split into identical 24 well chambers after 24 hours. After another 24 hours incubation, cells were treated with cycloheximide (100 ug/ml) in serum free media to arrest all protein synthesis, and groups of cells were analyzed at time points shown, when cell lysates were prepared and assayed for luciferase enzyme content. (A) The levels of NOTCH3-luciferase activity at the beginning of the time course did not differ significantly between any of the wildtype or mutant combinations. (B) Wildtype NOTCH3 ectodomain was more rapidly cleared than mutant NOTCH3 ectodomains. When cotransfections were performed with wildtype and mutant NOTCH3, proteins were cleared at an intermediate speed. (C) Quantification of a single time point (3 hours) is shown in a bar graph format to facilitate interpretation. The wild type protein is cleared most rapidly. Both CADASIL mutations slow clearance rate significantly. When mutants and wildtype protein were expressed together, there was an intermediate level of stability for R90C/wildtype protein. The C49Y/wildtype mixture was degraded at a similar rate as C49Y protein. Data is representative of three independent experiments. (*Significant compared to wildtype protein, #significant compared to all other groups; p<0.05.) Error bars represent standard deviations.

All proteins were expressed at similar activity levels prior to the addition of cycloheximide (Figure 5A). As expected, all proteins displayed a progressive decrease in protein expression over time after cycloheximide treatment. However, cells cleared the wildtype NOTCH3 fusion much faster than mutant NOTCH3 (Figure 5B and 5C). Specifically, decay of the R90C ectodomain was significantly impaired compared to wildtype, while C49Y ectodomain displayed a less severe reduction in clearance. When the wildtype and R90C mutant proteins were cotransfected in cells, the overall clearance of NOTCH3 ectodomains was reduced and displayed an intermediate between wildtype and mutant rates. When C49Y protein was cotransfected with wildtype NOTCH3, the clearance of NOTCH3 proteins adopted the slower clearance rate of the C49Y protein. These results suggest that the C49Y protein (but not the R90C protein) can dominantly influence the clearance rate of coexpressed NOTCH3.

### NOTCH3 Exerts a Dominant Negative Effects on Notch Signaling

Since both WT and mutant NOTCH3 dimerize with other NOTCH proteins and CADASIL mutations in NOTCH caused decreased solubility and clearance of NOTCH3 ectodomains, we hypothesized that increased NOTCH3 could affect Notch signaling. We tested how overexpressed full-length NOTCH3 affects Notch signaling by utilizing a well-established coculture system to measure the effects of expression of wildtype or mutant full-length NOTCH3 on Notch regulated smooth muscle genes.

**Figure 6:**
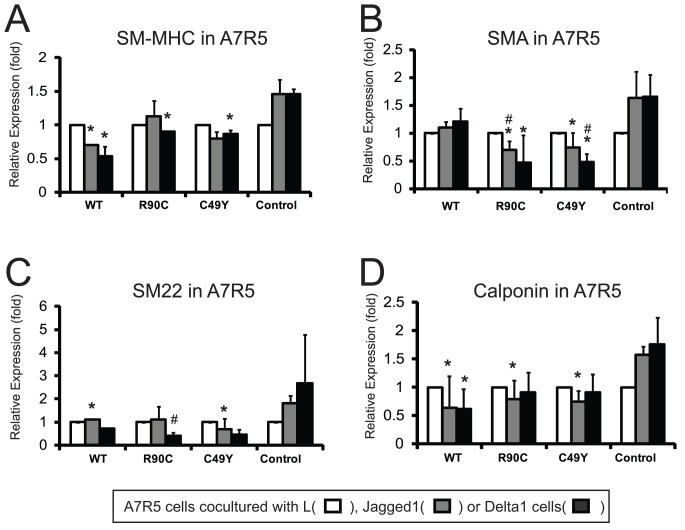
Inhibition of Notch-mediated smooth muscle marker expression by NOTCH3 transfection. A7R5 cells (n = 3 replicate wells per group) were cotransfected with NOTCH3 constructs shown. Control was empty vector pCMV-Sport6. After coculture with ligand expressing cells, mRNA levels of genes indicated were measured by quantitative RT-PCR using primers that detect rat sequences present in rat A7R5 cells but not mouse genes. Analysis was performed as noted in the methods and the display show the average level of gene induction in three independent experiments. * indicates differences between NOTCH3 transfected cells versus control cells and # denotes differences between mutant NOTCH3 and wildtype NOTCH3 transfectants (p<0.05). Error bars represent standard deviations.

A7R5 rat aortic smooth muscle cells express Notch1 and Notch3 mRNA, and, to a lesser degree, Notch4 transcripts (not shown). SM-MHC, SMA, SM22, and calponin were all upregulated in A7R5 cells by coculture with ligand producing cells (Figure 6). Transfection with full-length Notch3 (WT or mutant) clearly did not increase the expression of these genes; rather, NOTCH3 transfection inhibited ligand dependent increases in transcript levels in cocultures (rat smooth muscle mRNA was determined using species specific primers and qRT-PCR; Figure 6). We did not detect a consistent difference between the inhibitory effects of transfected full-length WT or mutant NOTCH3.

**Figure 7:**
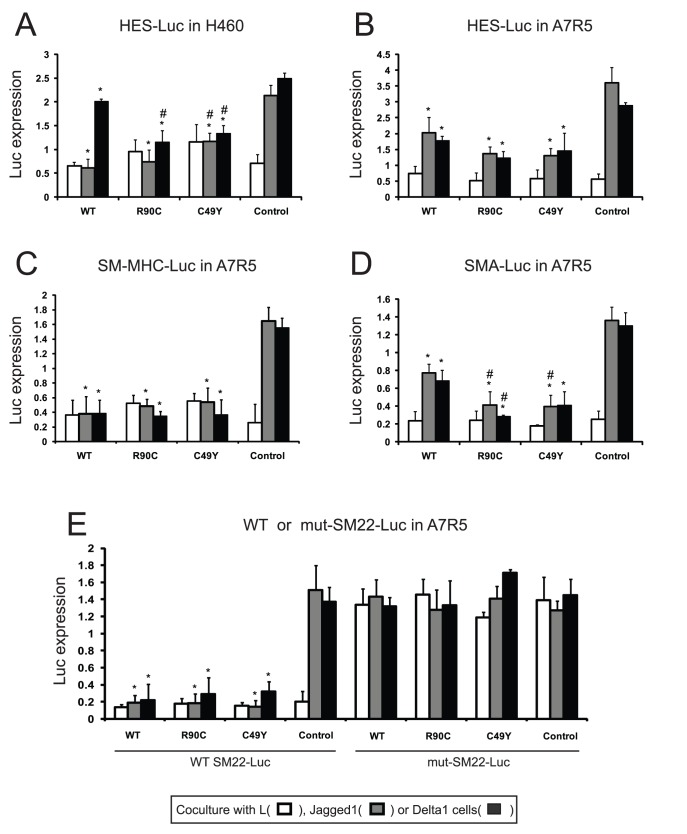
Inhibition of Notch mediated transcription by overexpression of full-length NOTCH3. Notch expressing cell lines H460 (A) or A7R5 (B–E) were cotranfected with luciferase reporters and either vector or full length NOTCH3 expression plasmids (n = 3 replicate wells per group). In panels A and B, we show effects of NOTCH3, mutants R90C and C49Y, and control vector on ligand stimulation of HES-luciferase. The effects of cotransfection of NOTCH3 on smooth muscle actin-luciferase (C), smooth muscle MHC-luciferase (D), SM22-luciferase (E, left group) and mutant SM22-luciferase (E, right group) are shown in the lower panels. After one day, cells were overlayed with L, Jagged1, or Delta1 expressing fibroblasts to assess the effect of Notch ligand stimulation. Luciferase assays were performed after 24 hours to quantify the level of Notch signaling. All ligand (Jagged1 or Delta1) stimulated activity was significantly suppressed by WT and mutant NOTCH3 transfection for every promoter tested (relative to control; p<0.05; the differences are not marked to simplify the presentation of data). Consistent and significant differences in Notch signaling were observed after coexpression of wildtype or mutant NOTCH3 in for H460 cells transfected with HES-luciferase (A) and for A7R5 cells transfected with all luciferase constructs except the mutant SM22 promoter. There were significant differences between the potency of inhibition of the mutant NOTCH3 proteins in selected groups. Each experiment was conducted three or more times; in each experiment, triplicate wells were analyzed. * indicates differences between NOTCH3 transfected cells versus control cells and # denotes differences between mutant NOTCH3 and wildtype NOTCH3 transfectants (p<0.05). Error bars represent standard deviations.

Notch signaling activates gene expression by derepressing CBF-1 responsive elements within promoter. To determine whether decreased smooth muscle mRNA levels could be due to decreased Notch activity, we examined the effects of full-length NOTCH3 overexpression on a prototypical CBF-1 regulated promoter by cotransfecting the HES-luciferase reporter into cells that were then cocultured with ligand producing cells. In transfected H460 cells (Figure 7A), which endogenously express only NOTCH3, cotransfection with wildtype or mutant full-length NOTCH3 resulted in inhibition of ligand-stimulated HES-luciferase activation. The transfection of either R90C or C49Y mutant NOTCH3 more potently inhibited Delta1-dependent NOTCH activation compared to wildtype NOTCH3. NOTCH3 overexpression failed to suppress the activity of constitutively active NOTCH3 lacking ectodomain sequences (NICD; Figure S2).

In A7R5 cells (Figure 7B), ligand-stimulated HES-luciferase activity was inhibited by wildtype and mutant full-length NOTCH3. There was a trend towards stronger suppression of Notch responses with expression of mutant NOTCH3 (compared to WT), but this did not reach statistical significance. Overall, both wildtype and mutant NOTCH3 repressed canonical Notch transcriptional function in a cell and ligand specific manner. The inhibition of the reporter construct was also seen after overexpression of NOTCH3 ectodomain (Figure S3).

Since levels of critical smooth muscle mRNAs were repressed by full-length NOTCH3 overexpression (Figure 6), we tested whether transfected NOTCH3 inhibits smooth muscle promoters normally targeted by Notch. In coculture assays, overexpression of all forms of full-length NOTCH3 resulted in inhibition of smooth muscle actin, smooth muscle myosin heavy chain, and SM22-luciferase constructs in A7R5 cells (Figures 7C–E). Although mutant NOTCH3 more potently inhibited the activity of the smooth muscle actin promoter (compared to wildtype NOTCH3; Figure 7D), there was no significant difference between the inhibitory properties of the wildtype and mutant NOTCH3 acting on the SM-MHC and SM22 promoters. All ligand-dependent promoter function was blocked by co-transfection with a DN-MAML peptide that has been shown to inhibit Notch signaling (not shown). Moreover, mutagenesis of the Notch-responsive element from the SM22 promoter construct fully blocked the inhibitory function of NOTCH3 overexpression (Figure 7E).

**Figure 8:**
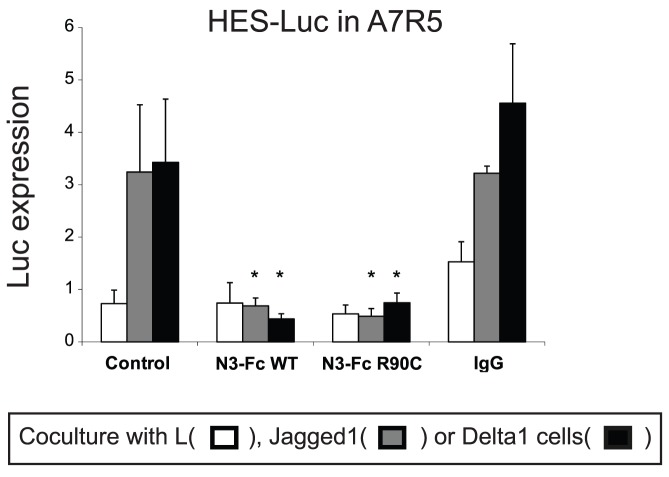
Inhibition of Notch signaling by extracellular NOTCH3 ectodomain. As in Figure 7, A7R5 cells (n = 3 replicate wells per group) transfected with HES-luciferase reporter (but not NOTCH3 cDNA) were cocultured with ligand producing or control cells. Experiments were performed in the presence of 1 ug/ml (3 nM) recombinant NOTCH3-Fc protein (wildtype or R90C mutant ectodomains fused to Fc). Control studies were done with no added protein and with purified human IgG to control for the presence of immunoglobulin in the NOTCH3-Fc preparation. * indicates differences between NOTCH3-Fc treated cells versus control cells (p<0.05). Experiments were done three times with similar results.

Co-transfection of NOTCH3 cDNA potentially causes aggregation of proteins in the intracellular compartment. To test whether secreted NOTCH3 ectodomain is sufficient to block Notch signaling, we prepared recombinant NOTCH3 ectodomain proteins purified from stable cell lines to determine their effects in Notch signaling. Both wild type and mutant NOTCH3 proteins added at nanomolar concentrations to the culture media were capable of potently inhibiting Notch signaling in A7R5 cells (Figure 8).

## Discussion

We report four novel findings regarding NOTCH3∶1) both wildtype and mutant NOTCH3 ectodomains form complexes with NOTCH1, 3, and 4 ectodomains via EGF-repeats; 2) CADASIL mutations decrease NOTCH3 detergent solubility; 3) CADASIL mutant protein interferes with cellular clearance of NOTCH3; 4) accumulated NOTCH3 represses Notch-stimulated expression of smooth muscle genes by interfering with promoter activation.

### NOTCH3 Ectodomain Protein Complexes

The appearance of GOM and granular NOTCH3 accumulation in blood vessels strongly suggests that vascular protein aggregation is a major pathological feature of CADASIL. Other studies [36], [38] have examined potential protein interactions between a number of other CADASIL mutants of NOTCH3 (R133C, C185R, C183R, and C455R). Here, we show novel, unequivocal evidence that both wildtype and two additional mutant NOTCH ectodomains (R90C and C49Y) form stable complexes within transfected cells and that the EGF-repeats are sufficient for protein complex formation. Importantly, we demonstrate for the first time that the NOTCH3 ectodomain is able to interact with ectodomains of NOTCH1 and NOTCH4, which are also expressed in the vascular wall. Our data demonstrate that NOTCH3 homomultimerization requires expression in the same cell, since lysate mixing did not result in multimer formation. Previous experiments have also suggested that NOTCH3 multimers form when expressed in cells [36], [38].

The significance of these protein interactions in CADASIL is two-fold. First, our interaction studies suggest that accumulated NOTCH3 could interfere not only with NOTCH3, but also with other NOTCH proteins in vessels. Second, the ectodomains of new binding partners NOTCH1 and NOTCH4 consist of primarily EGF-repeats; notably, all of the other known NOTCH3 partners (Jagged, Delta, the thrombospondins [42] and LRP1 [49]) contain EGF-repeats as well. Thus, it is probable that other EGF repeat containing proteins form complexes with NOTCH3. This possibility provides a framework to identify additional proteins that could interact with NOTCH3 in the pathogenesis of CADASIL.

### Solubility of NOTCH3 Mutants

We show new data that full-length mutant proteins are notably less soluble than wildtype proteins. A likely explanation for this is that mutant protein complexes form due to new protein-protein interactions, which has been suggested in an independent study of other CADASIL mutants [36]. Our experiments show that detergent resistance of the mutant protein was associated with the presence of intracytoplasmic perinuclear aggregates. In total, the results are consistent with the long held hypothesis that binding of NOTCH3 to other proteins form aggregates that could lead to disruptions on normal cell signaling required for smooth muscle homeostasis [36], [60], [61].

The relevance of perinuclear aggregates of NOTCH3 to CADASIL is unclear but similar staining has been observed by Karlstrom et al. [57]. Joutel et al. have found very similar intense aggregates of Notch3 in pericytes of the brain in juvenile transgenic mouse models of CADASIL [62], but not in earlier transgenic mice that failed to show white matter changes. The authors discussed the possibility that these aggregates were a consequence of overexpression of Notch3 in the vasculature, but, interestingly, rather than increasing over time, these aggregates disappear later in life despite continued expression of the protein in adult mice. The striking changes in distribution of these aggregates suggests that the localization of mutant Notch3 evolves over time, and raises the possibility that mutant Notch3 mutations may have different accumulation patterns and functional consequences over time. Overall, all studies which have expressed NOTCH3 in vitro, including the current one, have demonstrated significant intracellular expression. Despite significant efforts, we have been unable to express NOTCH3 at the cell surface without intracellular accumulation. Therefore, an important caveat to our study, and others, is that the effects of NOTCH3 overexpression on signaling may be a result of either effects inside the cell, or, potentially at the cell surface. In experiments reported in this study, we were able to inhibit global Notch signaling with purified, recombinant NOTCH3 ectodomain applied at nanomolar concentrations. Thus, NOTCH3 protein expressed outside the cell is sufficient to interfere with cell signaling.

Since we are able to control the solubility and quantity of Notch3 aggregates by altering cell culture density, this culture model of NOTCH3 expression may be useful in defining the regulation and significance of perinuclear Notch3 accumulation. The finding that low density cell plating results in increased aggregation and decreased solubility of NOTCH3 is consistent with prior findings that in CADASIL, protein accumulation accompanies dissociation between smooth muscle cells. While a causal connection remains speculative, further experiments using this model system to understand the molecular basis of this finding may shed light on the pathways towards NOTCH3 accumulation in CADASIL.

### Mutations in NOTCH3 Affect Clearance of Protein

Using a quantitative assay of protein clearance, we show that mutations in NOTCH3 significantly inhibit NOTCH3 ectodomain protein clearance. Takahashi and colleagues [38] have also shown that NOTCH3 mutations markedly slow the rate of clearance of two independent mutants on NOTCH3; therefore, the impairment of clearance of mutated NOTCH3 is likely to be a general property of CADASIL proteins. Since WT and mutant proteins were both labeled with luciferase, these experiments do not definitively identify the protein species whose clearance is altered. Nevertheless, we can conclude that the presence of mutant protein sequences can dominantly slow NOTCH3 turnover, which supports the hypothesis of Joutel et al. [58] that the accumulation of NOTCH3 in CADASIL is the result of impairment of protein clearance and not increased production. We further submit, based on biochemical studies above, that the inhibited clearance may be caused by changes in solubility resulting from both homotypic and heterotypic complexes involving NOTCH3-binding proteins such as NOTCH1 and NOTCH4.

We investigated whether mutants could dominantly affect other NOTCH3 ectodomain molecules by cotransfecting wildtype and mutant NOTCH3-luciferase fusions. Our new results indicated that the C49Y mutant did indeed dominantly affect clearance of other forms of NOTCH3, but that the R90C mutant did not. It remains to be tested whether dominant inhibition of clearance could be a more widespread property of CADASIL mutants and whether patients with mutations that dominantly affect NOTCH3 clearance have more severe disease and NOTCH3 accumulation.

### Excessive Full-length NOTCH3 Represses NOTCH Signaling

Full-length NOTCH3 overexpression could be expected to increase potency of Notch signaling. However, our studies soundly demonstrate that NOTCH3 overexpression dominantly suppresses Notch signaling in cells, including an arterial smooth muscle line. Both wild type and mutant NOTCH3 failed to activate smooth muscle promoters; but, instead, NOTCH3 overexpression suppressed Notch signaling; there was increased inhibition with mutant NOTCH3 in several assays, but this was not found with all mutants or promoters.

Mutant NOTCH3 was able to inhibit signaling more strongly than wildtype NOTCH3, but this was not a consistent finding. Based on the increased ability to inhibit clearance and induce protein aggregation, mutants could in fact be more potent in inhibiting NOTCH signaling, but due to a “floor effect” in our signaling assay, this could not be experimentally discerned. Indeed, recent studies of purified NOTCH3 ectodomains have demonstrated that mutant (but not WT) NOTCH3 proteins spontaneously form very large aggregates [37]. It is thus likely, since inhibition was seen with transfection of cDNA encoding the ectodomain (Figure S3) or application of purified NOTCH3 ectodomain (Figure 8) that the inhibitory effects we observe are due to competitive inhibition through ectodomain interactions. Lastly, because the activation of the NOTCH pathway by transfection of NICD from NOTCH3 was not affected by NOTCH3 overexpression, inhibition of Notch signaling most likely requires ectodomain interactions.

Thus, these cell studies may be consistent the following sequence of events in CADASIL: 1) mutations in NOTCH3 lead to decreased solubility; 2) NOTCH3 accumulates; 3) NOTCH3 accumulation leads to selective impairment of Notch signaling and gene activation. Since multiple Notch proteins are expressed in A7R5 cells, these studies also demonstrate that high levels of NOTCH3 likely inhibit multiple Notch isoforms.

Dominant negative effects of mutant NOTCH3 have been tested in only one other in vitro study, which failed to find suppressive effects of the R90C and C428S NOTCH3 mutants in cultured cells [8]. However, the C428S mutation, which causes impaired interactions with ligands, was found later to have partial dominant negative function in mice [61]. It is possible that our observations of signal inhibition were of greater magnitude because of differences in reagents and an experimental paradigm which favors detection of cross inhibitory complexes (longer expression period and avoidance of trypsin with EDTA). Another potential explanation for the differences in our results is that overexpression systems were used in these studies which enable very high levels of expression. Finally, our results differ in that added NOTCH3 was used to assess function of endogenous NOTCH proteins expressed at physiological levels in cells.

Previous investigators also did not detect a dominant negative effect of mutant Notch in the R90C transgenic mouse model [60]. But, it is difficult to compare this study to ours, since arteries of these mice likely did not express levels of Notch3 that we were able to achieve in vitro. In addition, our studies using multiple independent smooth muscle promoter luciferase reporters offer a new opportunity to examine relevant smooth muscle targets with improved quantitation compared to in vivo measurements in mice expressing a chromogenic reporter, which could miss modest changes in Notch activity.

In sum, the available data suggests that high levels of Notch3 expression in smooth muscle cells potently blocks Notch signaling. The consequences of global Notch inhibition in smooth muscle cells could be functionally significant, since marked arterial changes in mice with global smooth muscle Notch inhibition have been noted by Proweller [63]. These dominant negative effects could be clinically important, since Monet-Lepretre et al. have demonstrated that hypomorphic NOTCH3 mutations that lead to loss of NOTCH function result in more severe cerebral white matter changes on MRI in CADASIL patients [61].

## Supporting Information

Figure S1 Control experiments that demonstrate negative and positive interactions between NOTCH3 and fragments of the protein TSP2. We show two examples of fragments of TSP2 cotransfected with Notch3 and immunoprecipitated. (A) In the first pairwise interaction test, NOTCH3-HA and TSP2-myc tagged proteins were produced in abundance, but did not coprecipitate. (B) In the second pairwise interaction, another domain of TSP2 was shown to interact with Notch3. These data also demonstrate that HA and myc tags do not mediate protein-protein interactions.
(EPS)

Figure S2 Full-length NOTCH3 expression does not inhibit NICD-driven HES-luciferase activity. H460 cells were co-transfected with (a) full-length NOTCH3 (or vector), (b) NICD3 (or vector), and (c) HES-luciferase and Renilla-luciferase. After one day, HES-luciferase activity from cells (normalized to Renilla activity) was measured. Basal reporter levels were inhibited by all three full-length NOTCH3 constructs. Constitutive activation of the reporter by NICD was not affected by WT or mutant NOTCH3. This suggests that overexpressed NOTCH3 reduces activation of NOTCH3 signaling through ectodomain interactions, since NICD is missing the NOTCH3 ectodomain. * represents significant differences compared to N3-ICD groups. # represents significant differences between vector and NOTCH3 expressing cells (p<0.05; without N3-ICD cotransfection). Error bars represent standard deviations.
(EPS)

Figure S3 Inhibition of Notch mediated transcription by overexpression of NOTCH3 ectodomain A7R5 cells were cotranfected with HES luciferase reporter and either vector or NOTCH3 ectodomain expression plasmids. After one day, cells were cultured with L, Jagged1, or Delta1 expressing fibroblasts to assess activity resulting from Notch ligand stimulation. Luciferase assays were performed after an additional 24 hours and were normalized to co-transfected Renilla luciferase activity. All ligand-stimulated ectodomain groups exhibited significant inhibition of luciferase activity relative to matched control transfected cells (* p<0.05). Error bars represent standard deviations.
(EPS)
